# Electricity and the Processes of Life

**Published:** 1895-11-09

**Authors:** 


### Electricity and the Processes of Life.

The relation between electricity and those hidden processes of cell activity whose outward manifestations we recognise as the signs of life has always been a matter of the greatest interest. Unfortunately, its investigation has also been a matter of the greatest difficulty. The cells of which a large animal is built up are so connected with each other, so inter-related, and so buried in the mass, that the effect of exposing them to a current of electricity can only be judged of by very remote effects. Even the very fact of the influence of galvanism on vital processes, except at the points of entry and exit, has been doubted. Experiments which have been made, however, upon freely floating organisms are very suggestive, showing that the passage of even a steady current through them and the water in which they float—burying them, in fact, within a current —produces some change which is so far appreciated that they are driven to accommodate themselves to it. According to Dr. Augustus Waller,* if a galvanic current be passed through a bath containing paramœcia in sufficient abundance a curious sight is observed. When contact is made the whole crowd of paramœcia fall into order with their noses towards the kathode, and begin to swim towards it in converging curves, while if the current be reversed the crowd breaks up, all its units turn round and begin to swim away, as if of one mind, from the new anode to the new kathode; clearly these creatures are more "comfortable," if one may use the term, when swimming with the electric current than the reverse way. This, however, is not a general law for all micro-organisms, for some tend to swim against the current, and others again to place themselves at right angles to it. In a galvanic bath containing a mixture of ciliated and flagellated protozoa, while no current is passing these creatures swim about in all directions in a perfectly indifferent manner, but directly contact is made they divide themselves into two distinct armies, so to speak, which assemble on the two banks; "ciliata to the kathode, flagellata to the anode, seems to have been their *mot d'ordre*," and on reversing the current they immediately change places.

If in such a case we may speak of likes and dislikes, it is clear that each micro-organism is affected by the current in its own way, and is led to exercise a choice, and seek one pole or the other. So much for a free cell, which can move in any direction, but in regard to one which is tied fast, some similar influence must take place, and it is not difficult to believe that according to the direction of a current passing through it, may be the ease or difficulty with which it performs its function.

* "Science Progress," October, 1895.

In regard to more complex free-floating organisms the same is found to be true. Much as cats are more comfortable when stroked the right way than the wrong, and in fact will often get up and move away when stroked from tail to head, so it would seem that tadpoles dislike being "stroked" the wrong way by electricity. An experiment is described by Dr. Waller. In a lantern bath were a number of fresh tadpoles, moving more or less leisurely and jostling each other in all directions. On sending through it a current of electricity, he says "the commotion is amazing, the tadpole community seems to have gone mad, a writhing mass is all that can be distinguished; but the disturbance does not take long to subside, and now all the tadpoles are fixed as if at attention, heads to anode, viz., traversed by a current from head to tail, stroked down the right way."

It can also be shown that if the current is turned on very cautiously to a degree short of making the tadpoles face about, those which happen to be lying in such a direction that it passes through them from head to tail lie perfectly still, while those which lie the other way wag their tails; clearly the whole organism reacts differently according as the current goes in one direction or the other. If two tadpoles happen to be lying in the bath in opposite directions, by cautiously reversing the current the tadpoles may be made alternately one or the other to wag their tails. Of course in such complex creatures as tadpoles this reaction is not due to the effect of electricity upon individual cells, but depends on the presence of the spinal cord, as may be shown by experiment, for a piece of a tail long enough to contain a bit of spinal cord will tremble when the current is turned on, while a shorter piece is not affected.

These experiments are then sufficient to suggest that to be bathed in a galvanic current may be by no means so immaterial to the proper functionisation of the body as some people have imagined. If freely moving organisms are so affected as to swing round in response to the current, it is hard to believe that those embedded cells which cannot swing are any the less affected, and it is open to us to believe that they will perform their functions all the less perfectly from their inability to conform to their new surroundings. In relation to this it is not without interest to bear in mind the assertions continually made by many people as to the distressing effect upon them of what is termed thundery weather, when the relation between the atmospheric and the earth potential is reversed, and when, therefore, the direction of the current discharging through our bodies is abnormal.

